# Seeking a common human factors language for the development and testing of injection devices

**DOI:** 10.1080/10717544.2025.2541660

**Published:** 2025-08-07

**Authors:** Megan O. Conrad, Molly E. Laird, Mary Beth Privitera, Melissa R. Lemke, Molly Follette Story

**Affiliations:** a Mechanical Engineering, University of Detroit Mercy, Detroit, MI, USA; b HS Design, Morristown, NJ, USA; c Biomedical Engineering, University of Cincinnati, Cincinnati, OH, USA; d Human Ability Designs, Detroit, MI, USA; e Human Spectrum Design, LLC, San Carlos, CA, USA

**Keywords:** Pen injectors, autoinjectors, pre-filled syringe, task analysis, user interface, DDCP, human factors

## Abstract

Injection devices include drug–device combination products incorporating a needle for subcutaneous medication delivery. Often utilized by patients and caregivers, it is important for injection device user interface (UI) design to be intuitive to use outside of a traditional healthcare setting. To ensure safety and efficacy, the FDA requires a human factors (HFs) assessment as part of a new drug application (NDA) for new drug products and through the abbreviated new drug application (ANDA) pathway for generic drug approval. Despite the importance of defining injection device features as part of the HFs assessment, no comprehensive list of related definitions has previously been established. This paper compiles such definitions creating a common language for use in HFs assessments. Specifically, injection device classifications and characteristics are defined and then related to UI features and common tasks required for successful devices use. Information presented can be applied during device development and testing creating a common language for engineers, designers, and regulators. The definitions may be especially impactful for the approval of generic injection devices as the ANDA pathway for generic drugs requires a UI comparison of the proposed generic device to that of the existing reference listed drug.

## 1. Introduction

The US Food and Drug Administration (FDA) requires drug manufacturers intending to bring new drug–device combination products to market to prove both efficacy of the drug product while also providing evidence that the device is safe and effective at reliably administering medication. As defined by the FDA in 21 CFR 3.2(e), combination products are composed of two or more regulated components (e.g. drug and device, biologic and device) (CFR 2023).

Of the nine different types of combination products identified by the FDA, injection devices most commonly fall within the following three categories:

- *Convenience kit or co-package*: Drug and device are provided as individual constituent parts within the same package.
- *Prefilled drug delivery device/system*: Drug is filled into or otherwise combined with the device AND the sole purpose of the device is to deliver that drug.
- *Prefilled biologic delivery device/system*: Biological product is filled into or otherwise combined with the device AND the sole purpose of the device is to deliver that biological product.

Human factors (HFs) testing of the above products is especially important as combination products are primarily self-administered by patients. To verify a device can be safely and effectively self-administered, HF testing is conducted to assess the interaction of the intended users with the device’s user interface (UI). A thorough HF assessment considers usability of the UI design features alongside contextual factors including user characteristics and potential use environment(s).

For FDA approval of a new combination product, HF testing of the product is required as part of validation testing and submitted through the new drug application (NDA) regulatory pathway (FDA 2016). A proposed generic combination product must undergo a comparative HF process of the product against its reference listed drug (RLD) for approval through the abbreviated new drug application (ANDA) pathway (Federal Register 2017; Abbreviated New Drug Application (ANDA) 2022). The purpose of the comparative HF analyses is to demonstrate the proposed device used with the proposed generic drug is as safe and usable as the device used with the approved RLD.

A key component of the ANDA’s HF process requires comparison of any UI design changes and evaluation of whether the changes in UI design might affect the safety and effectiveness of device use. While FDA has issued draft guidance related to the HF evaluation of generic drug products (Federal Register 2017), the process of comparing device designs could be enhanced by having a set of common terms and definitions for categorizing and describing the components and attributes of these combination products.

Injection devices typically incorporate a needle to deliver medication subcutaneously (under the skin) and are designed to be easily usable by the intended users, including patients and healthcare professionals. A wide range of products are administered through injection devices, including both drugs and biologics. While some injection device components have been identified individually, no comprehensive list exists defining the components and attributes of all categories of combination products and the tasks involved in injection device use. Thus, the purpose of this paper is to provide a comprehensive list of terms that will facilitate identification and comparison of various types of injection devices as well as improve communication among all stakeholders. The information presented can be used during UI development, HF testing, regulatory submission, and analysis of use-related post-market problems.

## 2. Definitions

The definitions outlined in this paper are compiled from several sources, including government, industry, and other reference documents. In some cases, definitions were modified for specificity of the injection device or were created based on the authors’ expertise. If a definition does not include a citation, it was drafted by the research team based on the team’s combined knowledge and experience conducting HF studies as industry consultants, practitioners, academic researchers, and regulatory reviewers.

### 2.1. Injection device categories

Injection devices can be separated into three primary categories: pre-filled syringes, pen injectors (or injector pens), and auto-injectors. While all three device types administer the drug with which they are prefilled, differences between the device types relate to the primary container in which the drug is packaged and the mechanisms used to deliver the drug.

*Prefilled syringe*. A disposable syringe that is prefilled with a dose of the medicinal product (i.e. drug or biologic) to be injected by manually pressing the plunger rod into the syringe barrel.
*Pen injector -or- injector pen* (US Food and Drug Administration 2025). A device that provides a method of injecting an accurate dose of medicinal product from a medicinal cartridge, reservoir, or syringe through a manually-attached and -inserted, single-lumen needle.
*Auto-injector* (AUTO-INJECTOR 2025). A device that delivers a single, preloaded dose of a drug that typically consists of a spring-loaded syringe and a mechanism that administers the dose by automatically depressing the syringe plunger when the device mechanism is activated.

### 2.2. Injection device characteristics

Injection devices can be classified by the following characteristics: usage, dosage, reusability, activation mechanism (for auto-injectors), and locking mechanism. The following definitions are adapted from the FDA’s guidance document, technical considerations for pen, jet, and related injectors intended for use with drugs and biological products (U.S. Department of Health and Human Services 2013).

#### 2.2.1. Usage

*Single-use*. Device loaded with a single dose of drug/biologic; entire injector (including primary container) is discarded after single use.
*Multi-use*. Device loaded with multiple doses of drug/biologic; injector is used more than once for a single patient (to be labeled ‘for single patient use’).

#### 2.2.2. Dosage

*Fixed-dose*. Delivers the same dose each time for the number of injections specified in the labeling.
*Adjustable/variable-dose*. Delivers manually set dose(s).

#### 2.2.3. Reusability

*Disposable*. Injector with integral primary drug container is discarded after the drug container is empty.
*Reusable*. Injector with replaceable primary drug container closure is used multiple times and discarded when device reaches its end of life.

### 2.3. Definitions specific to pre-filled syringes

*Safety mechanism.* Mechanism built into a syringe that covers the needle after the syringe is used to reduce the occurrence of needlestick injuries; in some models, the mechanism rotates or slides over the needle and in other models the mechanism retracts the needle into the syringe barrel.
*No safety mechanism*. Syringe has no safety mechanism, and the needle is exposed before and after the syringe is used; typically, not used in professional healthcare settings.

### 2.4. Specific characteristics of auto-injectors

Auto-injectors have additional features due to the more complex nature of the device. These features include mechanisms to deliver the drug/biologic automatically when the device is activated, and some have a locking mechanism to prevent accidental activation.

*One-step activation* (U.S. Department of Health and Human Services 2013). Automatic injection starts when the user takes an action to activate the device, e.g. pushes the needle end of the device down on the injection site or pushes a button after placing the device on the injection site.
*Two-step activation* (U.S. Department of Health and Human Services 2013). Automatic injection starts when the user takes two actions to activate the device, e.g. first pushes the needle end of the device down on the injection site and then presses a button (e.g. on the distal end).
*Needle shield activation*. Automatic injection starts when the user presses the needle shield against the injection site hard enough to fully retract the needle shield.
*Button activation*. Automatic injection starts when the user presses a button after inserting the needle into or placing the needle end of the device on the injection site.
*Locking mechanism* (U.S. Department of Health and Human Services 2013). Safety feature that prevents accidental device activation; the user unlocks the locking mechanism before activating the device to administer the injection.

## 3. UI features of injection devices by category

Based on an evaluation of current injection devices on the market, the following summarizes the most common combination of device characteristics for each device category. Certain categories of devices typically have specific combinations of characteristics; for this paper, devices with common combinations of characteristics will be referred to as ‘platforms.’ The most common platforms observed within device categories include:

Pre-filled syringe:

- Single use, fixed dose, disposable;
- Single use, fixed dose, disposable with safety mechanism.

Pen injectors:

- Multiple use, adjustable dose, disposable;
- Multiple use, adjustable dose, reusable;
- Multiple use, fixed dose, disposable.

Auto-injectors:

- Single use, fixed dose, disposable with 1-step activation.
- Single use, fixed dose, disposable with 2-step activation.

**Figure 1.**
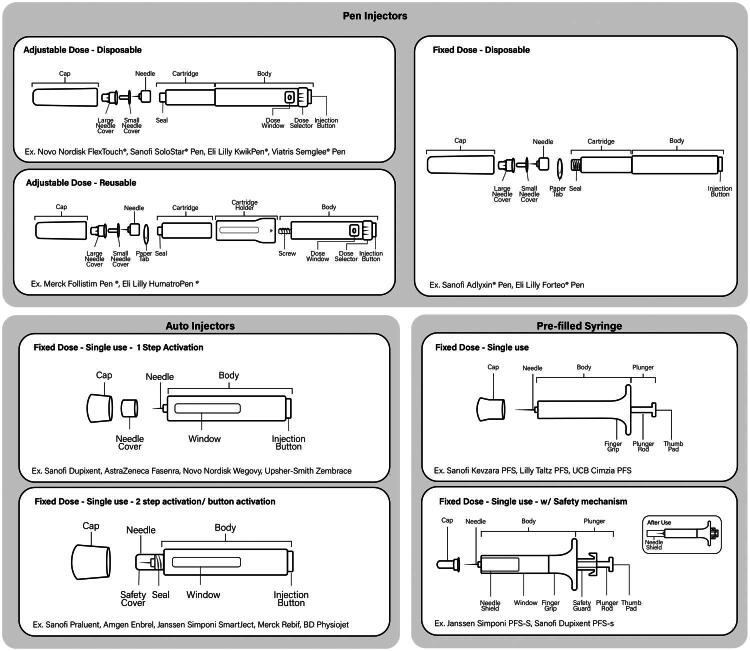
Representation of device UI features by injection device category and characteristics. Each image depicts common features of the device type and lists examples of devices currently on the market.

For each platform, these device characteristics drive UI design based on intended function and user needs. For example, all devices tend to have an injection needle, cap, body, etc. However, a dose dial is only necessary when adjustable dosing is desired. Figure 1 provides a visual representation of each common device type with the common UI characteristics of the platforms that fall within each device type. The figure also identifies currently marketed examples of these platforms.

## 4. Defining injection device tasks for HF assessments

Injection device UI design is driven by intended use, and the specific tasks required for drug administration via an injection device are driven by UI design features. At a high level, many of the use related tasks will remain the same between injection device types; however, differences in UI design can affect the subtasks involved.

For HF evaluations, a task analysis is a method for defining the specific steps required to meet a user’s goal. When applied to medical devices, task analyses can be an important design tool and can be used as a framework for associating UI design features with potential use-related risks (Kalita and Lemke 2019). Table 1 provides a baseline task analysis featuring common tasks and describes possible subtasks for each injector category. The list of tasks and subtasks was compiled from a variety of published usability studies evaluating pre-filled syringes (Hill et al. 2016; Dupixent 2025; MSKCC 2025), pen injectors (Lange et al. 2014; Lange and Nemeth 2018; Saunders et al. 2018; Klonoff et al. 2021; Lange et al. 2021), and auto-injectors (Edwards, Edwards, Davis, et al. 2015; Edwards, Edwards, Simons, et al. 2015; Domanska et al. 2016; Travanty et al. 2018; Benbadis et al. 2020; Serrecchia et al. 2020).

**Table 1.** Standard tasks and potential subtasks associated with various injection device platforms.

		Injector category						
		Multiple use			Single use			
		Pen			Auto-injector		Syringe	
Task	Subtasks *may* include:	Adjustable dose, disposable	Fixed dose, disposable	Adjustable dose, reusable	Fixed dose, disposable, button activated (2-step)	Fixed dose, disposable, needle shield activated (1-step)	Fixed dose, disposable	Fixed dose, disposable with safety system
1. Pre-delivery	Retrieve device, remove packaging, check expiration date, inspect device, inspect medication liquid, wash hands	x	x	x	x	x	x	x
2. Replace drug cartridge	Remove pen cap, unscrew old cartridge holder, remove old cartridge, slide new cartridge into holder, twist/screw holder onto pen			x				
3. Prep injection site	Select an injection site, clean with alcohol	x	x	x	x	x	x	x
4. Attach needle	Remove outer cap, wipe device seal, remove tab from needle, screw needle into pen	x	x	x			x	x
5. Prime needle	Hold device pointing up, tap pen to move air bubbles upward, push injector button or plunger to prime needle	x	x	x			x	x
6. Select dose	Turn the dosage knob to select dose, check dose window for correct dose	x		x				
7. Inject dose	*Pen*: Pinch skin, insert needle, push injection button, count to X seconds, remove needle	x	x	x				
	*Auto-injector*: Remove cap, pinch skin, place injector against skin, press down, press button, hold until medication administered, remove needle from skin				x	x		
	*Syringe*: Remove cap, pinch skin, insert needle, push plunger down, check syringe is empty, release pressure from plunger (retractable safety mechanism) – or – remove needle from skin (no guard) – or – remove needle from skin and move safety mechanism into place (manual mechanism)						x	x
8. Remove needle	Replace outer cap on needle, unscrew and remove needle			x				
9. Replace cap	Replace device cap	x	x	x				
10. Disposal	Discard needle and/or device in sharps container	x	x	x	x	x	x	x
11. Store pen	Store pen away from extreme heat or cold	x	x	x				

An x in the table indicates that the specific platform (column) requires the specified task (row) for correct use.

## 5. Discussion and conclusions

While development of combination products is increasing in the U.S. and throughout the world, stakeholders often use varying terms and definitions to describe the UI. This lack of consistency in terminology can introduce confusion and inefficiencies when attempting to collaboratively develop, define, and compare UI design attributes and differences during the design and subsequent regulatory review processes. These difficulties exist within NDA, ANDA, and other FDA regulatory submissions.

A common set of terms and definitions describing injection devices provides a consistent framework for both manufacturers preparing a device for FDA approval as well as regulatory reviewers responsible for assessing a device. Throughout design development these definitions can help developers understand how design features drive user interactions with injection devices including the tasks involved in the process of delivering an injection. Although the purpose of this paper aims to create a language enabling efficient communications between industry and reviewers, it is important to note that a common language could also be useful in patient-facing communications (e.g. labeling). Providing consistent terminology to the end user can minimize confusion regarding device use, particularly when end users are navigating adoption of a new device or generic version of a known combination product.

This framework can help improve clarity and quality of regulatory submissions which, in turn, can aid in efficiency of the review process. As an example, a sponsor submitting a proposed generic autoinjector through the FDA ANDA submission pathway can use this framework to describe and compare the UI characteristics related to usage, dosage, reusability, activation mechanism (for auto-injectors), and locking mechanism to ensure the regulatory reviewer understands the proposed design in comparison to the RLD. The sponsor also can ensure they consider the standard tasks and potential subtasks for each design, which will ensure the use-related risk analysis and any subsequent HF testing and evidence is presented to FDA with the required level of detail for FDA review. The framework also provides baseline information to ensure the design team considers critical parts of the UI and user interactions with the UI, which may be especially helpful to new and less experienced HF teams. This framework was developed with feedback from FDA stakeholders, and it is intended to reduce or eliminate known difficulties that exist within NDA, ANDA, and other FDA HF regulatory submissions.

## Data Availability

Data sharing is not applicable to this article as no new data were created or analyzed in this study.
